# Poisoning-related emergency department visits in children with autism spectrum disorder

**DOI:** 10.1186/s40621-022-00402-9

**Published:** 2022-12-21

**Authors:** Emma Cornell, Ashley Blanchard, Stanford Chihuri, Carolyn G. DiGuiseppi, Guohua Li

**Affiliations:** 1 Heilbrunn Department of Population and Family Health, Mailman School of Public Health, Columbia University, 722 W 168th St, New York, NY 10032 USA; 2 Department of Emergency Medicine, Columbia University Vagelos College of Physicians and Surgeons, 3959 Broadway, CHN-1-116, New York, NY 10032 USA; 3 Department of Anesthesiology, Columbia University Vagelos College of Physicians and Surgeons, 622 West 168th St, PH5-505, New York, NY 10032 USA; 4 Department of Epidemiology, Colorado School of Public Health, University of Colorado Anschutz Medical Campus, 13001 E. 17th Place, Mail Stop B119, Bldg. 500, Rm. W3138, Aurora, CO 80045 USA; 5 Department of Epidemiology, Columbia University Mailman School of Public Health, 622 West 168th St, PH5-505, New York, NY 10032 USA

**Keywords:** Autism spectrum disorder, Poisoning, Attention-deficit hyperactivity disorder, Intellectual disability

## Abstract

**Background:**

Autism spectrum disorder (ASD) is a complex neurodevelopmental condition, and its prevalence has increased markedly in the past two decades. Research indicates that people with ASD are at increased risk for premature mortality from injuries. Often, children with ASD are prescribed multiple medications, increasing their risk for intentional and unintentional poisonings. We examined the epidemiologic patterns of emergency department (ED)-treated poisonings in children with ASD and the association of ED-treated poisonings with ASD according to common co-occurring conditions.

**Methods:**

We analyzed data from the Nationwide Emergency Department Sample for 2016–2018 to estimate the frequencies of ED-treated poisonings among autistic children aged 1–20 years and adjusted odds ratios of ED-treated poisoning associated with ASD in the presence or absence of co-occurring attention-deficit hyperactivity disorder (ADHD) or intellectual disability (ID). The ICD-10-CM external cause-of-injury matrix was utilized to identify poisoning cases.

**Results:**

During 2016–2018, there were an estimated 523,232 ED visits in children with ASD aged 1–20 years, including 12,152 (2.3%) visits for poisoning. Of ED-treated poisonings in children with ASD, 73.6% were related to pharmaceutical drugs, such as psychotropic medications and prescription opioids, 16.6% were intentional, 36.5% were unintentional, and 47.0% were undetermined. Among children with ASD, those aged 5–9 had the highest odds of poisoning-related ED visits compared to all other age-groups (adjusted OR = 3.41; 95% CI 3.15, 3.68). The odds of poisoning for children with ASD were 59.0% greater than for their peers (adjusted OR = 1.59; 95% CI 1.53, 1.66) and varied significantly with age and co-occurring ADHD or ID.

**Conclusions:**

Children with ASD are at a significantly increased risk of poisoning, particularly among those aged 5–9 years. Co-occurring ADHD or ID with ASD further increases the risk of poisoning. Interventions to reduce poisoning in children with ASD should prioritize the safety of prescription medications.

## Background

Autism spectrum disorder (ASD) is a neurodevelopmental condition characterized by persistent challenges in social communication and social interaction, and restricted and repetitive behavior patterns, that typically manifests early in child development (American Psychological Association 2021). In 2016, the Autism and Developmental Disabilities Monitoring Network reported the ASD prevalence among US children to be 1 in 54, a 9.3% increase from the previous estimate in 2014 (Maenner et al. 2020; Baio et al. 2018). Approximately 70% of US children with an ASD diagnosis receive some form of treatment including 6.9% receiving medication treatment and 20.3% receiving a combination of medication and behavioral treatment (Xu et al. 2019). Children with ASD often have additional co-occurring diagnoses such as attention-deficit hyperactivity disorder (ADHD), anxiety disorders, and mood disorders (Soke et al. 2018), potentially leading to additional medication treatment. Among children with ASD, 59.1% have a co-occurring diagnosis of ADHD and 31% have an intellectual disability (ID) (Salazar et al. 2015; Baio et al. 2018).

In addition to increased access to medications in people with ASD, there is mounting evidence that people with ASD are at increased risk for premature mortality from injuries and chronic health conditions (Guan and Li 2017a; Smith DaWalt et al. 2019; Blanchard et al. 2021). In the US, unintentional injury is the leading cause of death among children aged 1–20 (CDC 2021a). The risk of unintentional injury death is heightened among children with ASD, and compared to individuals without ASD, those with ASD are about three times as likely to die from an injury (Guan and Li 2017a). Increased risk of unintentional injury death among children with ASD may be explained in part by increased frequency of wandering or elopement, which occurs more frequently among children with ASD, and can result in accidental drowning or traffic injuries (Rice et al. 2016; Guan and Li 2017b; McIlwain and Fournier 2018). In addition, a number of risk factors associated with increased injury risk, such as male sex, behavioral diagnoses, and maternal psychopathology occur at higher rates in children with ASD (DiGuiseppi et al. 2018; Jain et al. 2014).

Poisoning among children aged 1–20 accounts for 14.3% of unintentional injury mortality and 7.7% of suicide deaths in the USA (CDC 2021b). The prevalence of intentional injuries among those with ASD, including self-injurious behaviors (SIBs) and suicidality, is over 200% higher than those without ASD (Kirby et al. 2019; Kalb et al. 2016; Blanchard et al. 2021). Over a quarter of people with ASD have co-occurring attention-deficit hyperactivity disorder, 20% have co-occurring anxiety disorders, and 11% have co-occurring depressive disorders. These diagnoses are associated with an increased risk of suicide and may contribute to the increased prevalence of self-harm in people with ASD (Lai et al. 2019).

Children comprise a disproportionately large percentage of all poisoning cases reported to the American Association of Poison Control Centers’ National Poison Data System (NPDS) in the US: in 2018, 44.2% occurred among children under 6 years of age, followed by teens (8.2%) and children 6–12 years (6.3%) (Gummin et al. 2019). Using the Nationwide Emergency Department Sample (NEDS) data for the year 2008, Kalb et al. (2016) found that emergency department (ED) visits for injuries to children aged 3–17 years with ASD were 2.5 times as likely to be associated with poisoning (OR 2.50, 95% CI 1.67–3.75, *p* < 0.001) as injuries to children without ASD. Younger children with ASD may be at increased risk for unintentional poisoning due to access to medications being used for their treatment. Elevated odds of poisoning were also found in children with ADHD or ID (Kalb et al. 2016; Agnafors et al. 2020). Given the high prevalence of ID and ADHD in children with ASD, it is important to assess the potential interactions between ASD and these two co-occurring conditions on poisoning risk to inform prevention strategies. In this study, we aimed to determine the association of ASD with ED-treated poisonings among children aged 1–20 years according to the presence or absence of co-occurring ADHD or ID from 2016 to 2018. We hypothesized that children with ASD were at increased risk of ED-treated poisoning and that there would be a positive interaction between ASD and ADHD/ID on poisoning risk (i.e., the joint effects would be more elevated compared to the sum of the individual effects).

## Methods

### Data source

Data for this study came from the 2016 to 2018 NEDS, part of the Healthcare Cost and Utilization Project sponsored by the Agency of Healthcare and Research Quality (AHRQ 2020). The NEDS is the largest all-payer ED database in the USA yielding national estimates of more than 30 million ED visits annually. The NEDS is a 20% stratified, single-stage cluster sample constructed by categorizing hospitals according to five strata: geographic region, location, teaching status, ownership, and trauma-level designation. Data elements compiled in the NEDS come from state inpatient databases (patients initially seen in the ED and admitted to the same hospital) and state emergency department databases (patients released from ED or transferred to other hospitals). Using the International Classification of Diseases, Tenth Revision, Clinical Modification (ICD-10-CM), the NEDS presents up to 30 diagnoses associated with each visit in addition to visit type, demographic, hospital, and regional characteristics. Analyses were limited to patients aged 1–20 years.

### Measures

Poisoning cases were identified using the CDC ICD-10 injury diagnosis matrix. Poisoning was further categorized according to intent, i.e., unintentional (T36.0X1-T65.6X1, T65.811, T65.831, T65.891, T65.91X), intentional (T36.0X2-T65.96X2, T65.812, T65.832, T65.892, T65.92X), and undetermined (T36.0X4-T65.6X4, T65.814, T65.834, T65.894, T65.94X), according to the CDC 2020 ICD-10-CM poisoning matrix. We further coded poisoning according to 16 types of drugs (T36-T65) and categories of poison, i.e., prescription opioids (T402-T404), psychotropic drugs (T43), illicit drugs (T400, T401, T405, T407-T409, T436), other pharmaceutical drugs (T50.X, T36-T39, T41-T49), and non-medicinal substances (T51-T65). Non-medicinal substances include alcohol, organic solvents, carbon monoxide, pesticides, and halogen derivatives. ASD cases were identified using ICD-10 code F84.0, ADHD cases were identified using ICD-10 code F90.X, and ID cases were identified using ICD-10 codes F70-F79 (ASHA 2021). Demographic variables analyzed include age, sex, regional location of emergency department, urbanicity, and insurance payer (Medicare, Medicaid, Private Insurance, Self-pay, and other). Race and ethnicity variables were not available for 2016–2018 data obtained from the NEDS database and were thus not included (AHRQ 2021).

### Statistical analyses

We computed the weighted total number of ED visits and poisoning-related ED visits according to age, sex, region, urbanicity, payor status, ASD, ADHD, and ID and presented estimated odds ratios (ORs) and 95% confidence intervals (95% CIs). Weighted logistic regression modeling was used to estimate ORs and 95% CIs, comparing poisoning among children according to their ASD status and co-occurring ADHD or ID. In addition, we calculated odds ratios of poisoning-related ED visits associated with ASD, according to intent, relative to ED patients without ASD. Multivariable logistic regression models were used to estimate the odds ratios of poisoning-related ED visits associated with ASD when adjusting for age, sex, region, urbanicity, payor status, ADHD, and ID. Potential interaction between ASD and ADHD or ID was assessed on the multiplicative scale by inspecting the interaction term and on the additive scale by computing the relative risk due to interaction (RERI), attributable proportion due to interaction (AP) and the synergy index (*S*) (interpreted as follows: *S* = 1 indicates no interaction, *S* > 1 indicates positive interaction or more than additivity, and *S* < 1 indicates negative interaction or less than additivity) (Knol et al. 2011). Weighted frequencies of poisoning and type and drug category were graphed according to ASD status. Unweighted analyses were included as sensitivity analyses. Statistical significance was set at 0.05 for two-sided tests. All analyses were performed in SAS, version 9.4 (SAS Institute Inc.).

## Results

From January 1, 2016, to December 31, 2018, the NEDS recorded a total of 263,949 poisoning-related ED visits in children aged 1–20 years, yielding an estimated national total of 1,136,817 poisoning-related ED visits in this population group based on the sampling weights. Of the poisoning-related ED visits, 39.1% were unintentional, 25.7% were intentional (including 0.3% of assaultive intent), and 35.2% were undetermined. Among children with ASD, 16.6% were intentional, 36.5% were unintentional, and 47.0% were undetermined poisoning-related visits. The proportion of ED visits that was related to poisoning varied significantly with demographic and clinical characteristics (Table 1). Specifically, ED visits for older children, girls, and children with ASD, ADHD, or ID were more likely to be due to poisoning (Table 1). Poisonings accounted for 2.3% of all ED visits in children with ASD, and 1.2% of ED visits in children without ASD, yielding a crude OR of 1.89 (95% CI 1.82–1.96). Compared to the Northeast region, ED visits of children in the Midwest, South, and West regions were more likely to be due to poisoning (Table 1). Privately insured children and children insured by Medicare were more likely to visit the ED for poisoning-related injuries compared to children insured by Medicaid or self-pay (Table 1).

**Table 1:** Weighted frequencies estimated odds ratios (OR) and 95% confidence intervals (CI) of poisoning according to demographic and clinical characteristics in children aged 1–20 years, weighted data from the Nationwide Emergency Department Sample 2016–2018

Characteristic	Number of ED visits	Number of ED visits for poisoning (%)	Crude OR (95% CI)	*p* value	Adjusted* OR (95% CI)	*p* value
*Age (years)*						
1–4	25,923,734	308,542 (1.19)	2.15 (2.12, 2.18)	< 0.0001	2.25 (2.22, 2.28)	< 0.0001
5–9	18,814,831	104,930 (0.56)	1.00		1.00	
10–14	16,539,466	177,986 (1.08)	1.94 (1.91, 1.97)		1.83 (1.80, 1.86)	
15–20	29,716,652	545,360 (1.84)	3.33 (3.29, 3.38)		3.16 (3.12, 3.20)	
*Sex*						
Male	44,234,677	485,643 (1.10)	1.00	< 0.0001	1.00	< 0.0001
Female	46,755,522	651,076 (1.39)	1.27 (1.26, 1.28)		1.22 (1.21, 1.23)	
*Region*						
Northeast	15,649,793	174,398 (1.11)	1.00	< 0.0001	1.00	< 0.0001
Midwest	21,794,869	293,857 (1.35)	1.21 (1.20, 1.23)		1.25 (1.23, 1.26)	
South	36,884,957	426,854 (1.16)	1.04 (1.03, 1.05)		1.11 (1.10, 1.13)	
West	16,665,064	241,707 (1.45)	1.31 (1.29, 1.32)		1.38 (1.37, 1.40)	
*Urbanicity*						
Rural	13,964,418	170,093 (1.22)	1.00	< 0.0001	1.00	< 0.0001
Urban	77,030,265	966,724 (1.26)	1.03 (1.02, 10.4)		1.05 (1.04, 1.06)	
*Payor*						
Medicare	439,283	5344 (1.22)	1.11 (1.04, 1.17)	< 0.0001	1.01 (0.95, 1.07)	< 0.0001
Medicaid	52,401,337	577,151 (1.10)	1.00		1.00	
Private Insurance	27,713,002	431,840 (1.56)	1.42 (1.41, 1.43)		1.35 (1.34, 1.36)	
Self-pay	7,230,319	79,382 (1.10)	1.00 (0.98, 1.01)		0.91 (0.90, 0.93)	
Other	3,077,233	40,833 (1.33)	1.21 (1.18, 1.23)		1.12 (1.09, 1.14)	
*Attention-deficit/hyperactivity disorder*						
No	89,588,147	1,082,202 (1.21)	1.00	< 0.0001	1.00	< 0.0001
Yes	1,406,535	54,615 (3.88)	3.30 (3.24, 3.37)		3.35 (3.29, 3.42)	
*Intellectual disability*						
No	90,910,396	1,133,683 (1.25)	1.00	< 0.0001	1.00	< 0.0001
Yes	84,286	3134 (3.72)	3.06 (2.84, 3.30)		1.74 (1.61 1.89)	
*Autism spectrum disorder*						
No	90,471,451	1,124,665 (1.24)	1.00	< 0.0001	1.00	< 0.0001
Yes	523,232	12,152 (2.32)	1.89 (1.82, 1.96)		1.59 (1.53, 1.66)	

*Adjusted for age, sex, and region, urbanicity, payor, ADHD, and ID

**Fig. 1:**
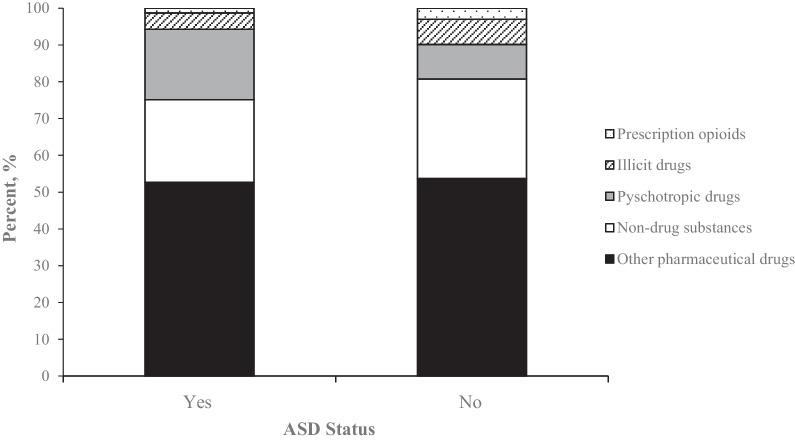
Percentage distributions of poisonings by autism spectrum disorder (ASD) status and drug category in children aged 1–20 years, weighted data from the Nationwide Emergency Department Sample 2016–2018

Pharmaceutical drugs accounted for 73.6% of poisoning-related ED visits in children with ASD, compared with 66.3% in children without ASD (*p* < 0.0001). Specifically, compared to children without ASD, poisonings in children with ASD were more likely to be related to psychotropic medications (22.7% vs 12.2%, *p* < 0.0001) and antiepileptic sedative-hypnotics (12.6% vs. 6.7%, *p* < 0.0001). In contrast, poisonings in children without ASD were more likely than those in children with ASD to involve prescription opioids (3.1% vs. 1.4, *p* < 0.0001) and illicit drugs (6.7% vs. 4.3%, *p* < 0.0001, Fig. 1) (Table 2).

**Table 2:** Percentage distributions of poisoning types by autism spectrum disorder (ASD) status in children aged 1–20 years, weighted data from the Nationwide Emergency Department Sample 2016–2018

Type of poisoning	ASD (*n* = 12,152) %	No ASD (*n* = 1,124,665) %
Systemic antibiotics	5.35	9.02
Systemic anti-infectives and antiparasitics	1.08	1.58
Hormones and their synthetic substitutes	4.34	3.71
Nonopioid analgesics, antipyretics, antirheumatics	5.63	12.79
Narcotics and hallucinogens	2.51	7.53
Anesthetics and therapeutic gases	0.61	0.30
Antiepileptic, sedative-hypnotics	12.61	6.70
Psychotropic drugs	22.68	12.21
Drugs affecting the autonomic nervous system	2.03	1.87
Systemic and hematological agents	7.20	7.66
Drugs affecting the cardiovascular system	7.09	2.41
Drugs affecting the gastrointestinal system	0.74	0.78
Drugs affecting smooth and skeletal muscles and the respiratory system	2.84	3.29
Topical agents affecting skin, mucous membrane, and ophthalmological, otorhinolaryngological, and dental drugs	1.51	1.81
Diuretics and other and unspecified drugs, medicaments, and biological substances	13.27	14.04
Other	10.50	14.31
Total	100.00	100.00

Relative to children without ASD, the estimated odds ratios of ED-treated poisoning associated with ASD increased significantly for each age-group, ranging from 1.45 (95% CI 1.30, 1.62) in children aged 1–4 years to 3.41 (95% CI 3.15, 3.68) in children aged 5–9 years, 2.13 (95% CI 1.98, 2.30) in children aged 10–14 years, and 1.86 (95% CI 1.74, 1.98) in children aged 15–20 years. However, the estimated odds ratios of ED-treated poisoning associated with ASD were similar for girls (OR = 1.96; 95% CI 1.82, 2.12) and boys (OR = 2.07; 95% CI 1.98, 2.17). The estimated odds ratios of ED-treated poisoning associated with ASD were highest for undetermined intent (OR = 2.53; 95% CI = 2.39, 2.67), followed by unintentional poisoning (OR = 1.76; 95% CI = 1.65, 1.88) and intentional poisoning (OR = 1.23; 95% CI = 1.12, 1.35).

Compared to children without ASD, ADHD, or ID, the estimated adjusted odds ratios of ED-treated poisoning were 2.08 (95% CI 1.99, 2.17, *p* < 0.0001) for children with ASD but without ADHD or ID, 3.44 (95% CI 3.37, 3.35, *p* < 0.0001) for children with ADHD or ID but without ASD, and 3.52 (95% CI 3.25, 3.80, *p* < 0.0001) for children with ASD and either ADHD or ID (Table 3). There was a significant negative interaction between ASD and ADHD or ID on ED-treated poisoning on both the multiplicative (*b* = − 0.67 95% CI − 0.76, − 0.58) and additive scales (RERI = − 1.09 95% CI − 1.33, − 0.83); AP = 0.37 95% CI − 0.49, − 0.27; *S* = 0.64 95% CI 0.57, 0.72). Results from weighted analysis and unweighted analysis were consistent (Appendix Table 4).

**Table 3:** Estimated odds ratios (ORs) and 95% confidence intervals (CIs) of poisoning associated with autism spectrum disorder (ASD) according to the presence of attention-deficit hyperactivity disorder (ADHD) or intellectual disability (ID) in children aged 1–20 years, weighted data from the Nationwide Emergency Department Sample 2016–2018

Co-occurring disorder status		Percentage of ED visits for poisoning	Crude OR (95% CI)	*p* value	Adjusted OR* (95%CI)	*p* value
Autism spectrum disorder	Attention-deficit/hyperactivity disorder or intellectual disability					
No	No	1.21	1.00		1.00	
Yes	No	2.09	1.75 (1.67, 1.83)	< 0.0001	2.08 (1.99, 2.17)	< 0.0001
No	Yes	3.85	3.27 (3.21, 3.34)	< 0.0001	3.44 (3.37, 3.51)	< 0.0001
Yes	Yes	3.47	2.94 (2.72, 3.17)	< 0.0001	3.52 (3.25, 3.80)	< 0.0001

*Adjusted for age, sex, region, urbanicity, and payor

## Discussion

Results from this analysis indicate that children on the autism spectrum were more likely to visit the ED for poisoning-related injuries when compared to children without a diagnosis of ASD. This is consistent with prior research demonstrating an increased likelihood of poisoning among children with ASD (Kalb et al. 2016). Children with ASD and ADHD or ID were even more likely to visit the ED for poisoning-related injuries. By further examining specific age, gender, intent, and substance-related differences in children with ASD, our results can inform targeted prevention strategies to reduce the incidence and prevalence of pediatric poisoning cases in this population.

The presence of a co-occurring diagnosis of ADHD or ID compounds the likelihood of poisoning-related injury among children with ASD. Compared to children without a diagnosis of ASD, ADHD, or ID, the estimated odds of being treated in the ED for poisoning among children with a diagnosis of ASD without ADHD or ID was lower than the OR for children with ASD and either ADHD or ID. The negative interaction between ASD and ADHD or ID on the risk of poisoning-related ED visits shows that the combined effects of the co-occurring disorders were lower than the net sum effects associated with the disorders. These results do not support our hypothesis of a positive interaction. Nevertheless, they suggest that children with ADHD or ID may have a heightened poisoning risk profile and etiology compared to children with ASD. This is consistent with previous research indicating that children with ADHD experience an independent risk of poisoning compared to children without a diagnosis of ADHD (Agnafors et al. 2020; Ruiz-Goikoetxea et al. 2018).

Pharmaceutical drugs represent the greatest poisoning risk for children with ASD, accounting for nearly three-quarters of poisoning-related ED visits. The high percentage of pharmaceutical-related poisonings for children with ASD is consistent with national poisoning trends in the general pediatric population, which have increased in the past decade, in part due to increased access to prescription medication at home, where the majority of poisonings occur (Spiller et al. 2013; Wynn et al. 2016). This higher percentage of pharmaceutical poisoning cases among children with ASD compared to children without ASD may reflect an increased access to prescription medications to manage ASD (Xu et al. 2019). In addition, given the documented presence of co-occurring diagnoses among children with ASD including other psychiatric diagnoses, epilepsy, and congenital heart disease, these children may also have increased access to medications prescribed to treat these conditions (Lukmanji et al. 2019; Sigmon et al. 2019; Soke et al. 2018). Psychotropic medications accounted for the largest percentage of poisoning cases by medication type (22.7%); these medications are used to treat anxiety, mood disorders, and ADHD, all of which have a higher incidence among people with ASD (Soke et al. 2018). Poisoning involving antiepileptic drugs and drugs affecting the cardiovascular system also occurred more frequently in children with ASD compared to children without ASD.

Children aged 5–9 with ASD had the highest odds of poisoning-related ED visits, followed by children aged 10–14. While other studies have examined the association between ASD and poisoning, they have not stratified results by age. Previous work examining the association between age and poisoning-related ED visits in the general pediatric population has found the highest odds of poisoning-related ED visits occurs in the 15–21 age-group followed by the 1–4 age-group (Kline et al. 2021). Particularly high rates of poisoning-related ED visits in school-age children with ASD may further substantiate the greater exposure to prescription medications early in childhood.

Among children with ASD, boys visit the ED disproportionately compared to girls, comprising over 78% of all ED visits. This is consistent with estimates that ASD is about 4 times as prevalent among boys than girls, due in part to underdiagnosis among girls (Maenner et al. 2020; ADDM 2021). Additionally, boys with ASD more frequently present to the ED, and thus our data are consistent with reported national prevalence trends in the general population (Liu et al. 2017).

Undetermined poisoning cases accounted for approximately 35.2% of cases, and the odds of an undetermined poisoning among children with ASD were 2.53 the odds of poisoning in children without ASD. The large percentage of undetermined intent may reflect misclassification in ICD coding for intent, and systemwide inability to accurately identify intention in poisoning cases among children with ASD, who may communicate differently than children without ASD. Our findings are consistent with previous research, which indicates that the majority of pediatric poisoning cases are unintentional or undetermined (Gummin et al. 2019).

Pediatric and ED providers should be aware of the increased likelihood of poisoning-related injuries among children with ASD, and take steps to educate caregivers and families on practical ways to reduce the risk of unintentional poisoning injury, particularly for boys aged 5–9 years. Increased provision of anticipatory guidance highlighting the importance of safe medication storage through lethal means counseling and provision of medication lock boxes or cabinet locks to families of children with ASD, may offer opportunities for clinicians and public health officials to intervene and prevent poisoning-related injuries. Continued emphasis on childproofing homes and utilization of child-resistant medication bottles through age twelve may be appropriate, as our findings suggest an increased risk of poisoning-related ED visits among children with ASD until age 12. Since our findings suggest psychotropic medications account for the largest percentage of unintentional poisonings among children with ASD, dedicated educational outreach efforts to psychiatric providers may help increase awareness about the importance of safety considerations and medication storage counseling to caregivers when prescribing psychotropic medications for children with ASD.

## Limitations

Our study has several limitations. First, the NEDS dataset utilizes encounter-level records. As a result, if a patient visits the ED multiple times in a year for poisonings, each encounter will be recorded as a separate case. In addition, evidence indicates that 87.5% of poisoning cases for children < 5 years, and 81.6% % of poisoning cases for children aged 6–12 years do not result in medical intervention or an ED visit (Gummin et al. 2019). Thus, the NEDS dataset is unable to capture a substantial proportion of poisonings in the pediatric population that do not result in an ED visit. It is possible that some children presented to the ED for a poisoning-related injury, but that the visit was not characterized as related to poisoning, resulting in an underestimation of the actual number of poisoning cases (Gummin et al. 2019). Similarly, documentation of a diagnosis of ASD, particularly in situations where there is an acute medical need for intervention, may not always occur, resulting in misclassification and possible underestimation of cases. Prior to 2019, the NEDS database did not collect race/ethnicity data for patient characteristics, excluding an important demographic variable that might highlight additional health disparities among children with ASD (AHRQ 2021). This dataset utilizes the gender binary to describe patients, and as such is unable to capture children who may identify outside of the traditional gender binary. Finally, determining intent associated with injuries in children is subject to interpretation, and is thus an imperfect variable to utilize. Particularly among children, parents often report the circumstances surrounding a child’s visit to providers, and their perspective may differ from the child’s perception of the event. In addition, children with ASD who may be non-verbal may be unable to communicate their intent to medical providers and caregivers.

## Conclusion

This nationally representative study of emergency department visits seeks to report the prevalence of pediatric poisoning cases among children diagnosed with autism spectrum disorder and describe the co-occurring diagnoses, toxic substances, intent, and demographic patterns associated with poisonings in this particular population. Among children with ASD, males, children aged 5–9, and children with co-occurring diagnoses of ADHD or ID were more likely to visit the ED for a poisoning-related injury. Pharmaceutical medications accounted for the greatest proportion of poisoning types in this population. Targeted interventions designed to educate families and caregivers about the importance of safe medication storage may be efficacious in reducing the risk of poisoning among children with ASD. Providing families with proactive safety planning tools may be advantageous for children with ASD and co-occurring ADHD or ID. Future studies should examine the impact of such interventions in this population, as well as additional sociodemographic factors such as geographic location, race, and insurance status to better comprehend the patterns of poisoning injury across the USA.

## Data Availability

The datasets generated and analyzed for this study are available in the NEDS repository, https://www.hcup-us.ahrq.gov/db/nation/neds/nedsdbdocumentation.jsp.

## Appendix

See Table 4.

**Table 4:** Frequencies and estimated odds ratios (ORs) and 95% confidence intervals (CIs) of poisoning by demographic and clinical characteristics in children aged 1–20 years, unweighted data from the Nationwide Emergency Department Sample 2016–2018

Characteristic	Number of ED visits	Number of ED visits for poisoning (%)	Crude OR (95% CI)	*p* value	Adjusted* OR (95% CI)	*p* value
*Age (years)*						
1–4	6,086,931	71,649 (1.18)	2.15 (2.12, 2.18)	< 0.0001	2.26 (2.22, 2.29)	< 0.0001
5–9	4,422,848	24,328 (0.55)	1.00		1.00	
10–14	3,873,816	41,134 (1.06)	1.94 (1.91, 1.97)		1.84 (1.81, 1.87)	
15–20	6,949,166	126,838 (1.83)	3.36 (3.31, 3.41)		3.19 (3.14, 3.23)	
*Sex*						
Male	10,372,158	112,978 (1.09)	1.00	< 0.0001	1.00	< 0.0001
Female	10,959,617	150,948 (1.38)	1.27 (1.26, 1.28)		1.22 (1.21, 1.23)	
*Region*						
Northeast	3,588,113	39,788 (1.11)	1.00	< 0.0001	1.00	< 0.0001
Midwest	4,827,000	64,083 (1.33)	1.20 (1.19, 1.22)		1.24 (1.22, 1.25)	
South	8,731,769	100,417 (1.15)	1.04 (1.03, 1.05)		1.11 (1.10, 1.13)	
West	4,185,879	59,661 (1.43)	1.29 (1.27, 1.31)		1.37 (1.35, 1.39)	
*Urbanicity*						
Rural	2,909,453	34,669 (1.19)	1.00	< 0.0001	1.00	< 0.0001
Urban	18,423,308	229,280 (1.24)	1.05 (1.03, 1.06)		1.05 (1.04, 1.07)	
*Payor*						
Medicare	100,106	1225 (1.22)	1.13 (1.06, 1.19)	< 0.0001	1.03 (0.97, 1.09)	
Medicaid	12,318,930	134,050 (1.09)	1.00		1.00	
Private Insurance	6,447,546	99,925 (1.55)	1.43 (1.42, 1.44)		1.36 (1.35, 1.37)	
Self-pay	1,723,958	18,837 (1.09)	1.00 (0.99, 1.02)		0.92 (0.90, 0.93)	
Other	714,981	9456 (1.32)	1.22 (1.19, 1.24)		1.13 (1.11, 1.15)	
*Attention-deficit/hyperactivity disorder*						
Negative	21,010,983	251,424 (1.20)	1.00	< 0.0001	1.00	< 0.0001
Positive	321,778	12,525 (3.89)	3.35 (3.28, 3.41)		3.39 (3.32, 3.45)	
*Intellectual disability*						
Negative	21,313,181	263,222 (1.24)	1.00	< 0.0001	1.00	< 0.0001
Positive	19,580	727 (3.71)	3.08 (2.86, 3.32)		1.76 (1.63, 1.89)	
*Autism spectrum disorder*						
Negative	21,210,169	261,136 (1.23)	1.00	< 0.0001	1.00	< 0.0001
Positive	122,592	2813 (2.29)	1.89 (1.82, 1.96)		1.59 (1.53, 1.65)	

*Adjusted for age, sex, and region, urbanicity, payor ADHD, and ID

## Abbreviations

ADHD: Attention-deficit hyperactivity disorder
ASD: Autism spectrum disorder
ED: Emergency department
ID: Intellectual disability
ICD-10-CM: International Classification of Diseases 10th Revision Clinical Modification
NEDS: National Emergency Department Sample
NPDS: National Poison Data System
OR: Odds ratio
US: United States
